# Research satisfaction among participants in a preclinical Alzheimer's disease trial

**DOI:** 10.1002/alz.71852

**Published:** 2026-09-22

**Authors:** Marina Ritchie, Kedir Hussen, Oliver Langford, Michael C. Donohue, Mary Sano, Joshua D. Grill, Paul Aisen, Reisa Sperling, Rema Raman

**Affiliations:** ^1^ Epstein Family Alzheimer's Therapeutic Research Institute University of Southern California San Diego California USA; ^2^ Icahn School of Medicine at Mount Sinai New York New York USA; ^3^ University of California, Irvine Irvine California USA; ^4^ Center for Alzheimer Research and Treatment Brigham and Women's Hospital, Harvard Medical School Boston Massachusetts USA

**Keywords:** clinical trial, participant feedback, preclinical AD, research satisfaction

## Abstract

**INTRODUCTION:**

Empirical data capturing participants’ experiences in preclinical Alzheimer's disease (AD) trials are limited. We examined participant responses on a Research Satisfaction Survey (RSS) from the Anti‐Amyloid Treatment in Asymptomatic AD Study.

**METHODS:**

Participants completed the RSS at week 0, 48, 108, 168, and 240. We examined potential differences in responses by baseline participant characteristics and trial completion status.

**RESULTS:**

RSS scores were high, with > 90% of participants responding positively to all satisfaction items. Across timepoints, altruistic motivations were most frequently affirmed, while study design elements were most frequently disfavored. Affirmation of social motivations increased over time. Participants who responded “not satisfied” at baseline to repeating their decision to enroll had higher odds of study discontinuation than those who responded “satisfied” (1.99; 95% confidence interval [CI]:1.24, 3.19).

**DISCUSSION:**

An RSS captures participant experiences in a preclinical AD trial, can guide decisions on study design features, and may identify those at risk for loss to follow‐up.

## Highlights

- Participants in the A4 Study were highly satisfied with their trial experience
- Baseline measures of research satisfaction may serve as a tool to assess risk of participant attrition
- Participants most frequently favored trial aspects associated with altruistic, personal, and social motivations
- Participants most frequently disfavored trial aspects associated with design and individual expectations/fears

## 1 BACKGROUND

A growing number of Alzheimer's disease (AD) trials test treatments at preclinical stages of the disease, prior to symptom onset.^1^, ^2^, ^3^ Many of these trials evaluate candidate treatments that have the potential to be disease‐modifying, and are therefore key to curbing the growing public health burden from AD. The success of these trials is contingent on the timely randomization of enrolled participants and maximum retention of participants as they directly impact the statistical power, risk of bias, and generalizability of study results.^4^, ^5^, ^6^, ^7^ Preclinical AD trials typically require long follow‐up duration to assess therapeutic efficacy, underscoring the need to identify effective strategies to support and maximize participant retention. Despite the importance of understanding factors that influence decisions around trial enrollment and continued participation, there are limited data on participant experiences in preclinical AD trials.^8^

In the present study, we sought to examine participant feedback within a global phase III randomized, controlled clinical trial setting among an understudied population of cognitively unimpaired participants with AD. To address this objective, we used data from the Anti‐Amyloid Treatment for Asymptomatic AD (A4) Study, the first completed preclinical AD trial to systematically incorporate a Research Satisfaction Survey (RSS).^9^ The RSS is an eight‐item, self‐reported questionnaire administered longitudinally over the course of the trial to assess overall participant satisfaction with the trial, characterize the most favored and disfavored aspects of trial participation, and identify areas perceived as needing improvement.^9^ The RSS used in the A4 Study was adapted from a tool used to assess satisfaction with clinical services and has been found to identify modifiable features that could improve patient experience.^10^, ^11^, ^12^ Although the investigational treatment did not meet its primary endpoints in the A4 trial, the study randomized 1169 participants and retained 830 (71%) individuals over 4.5 years, providing a unique opportunity to examine factors associated with trial engagement and early discontinuation in a preclinical AD population.^13^ By assessing baseline characteristics associated with RSS responses over time and comparing them between those who complete the trial (“completers”) and those who discontinue early (“non‐completers”), we aimed to identify specific aspects of the trial that shaped participant experience.

## 2 METHODS

### 2.1 Design and data

We present results of secondary analyses of de‐identified data from the double‐blind phase of the A4 Study (ClinicalTrials.gov identifier: NCT02008357), conducted from 2014 to 2022.^9^ Participants randomized in the study were ages 65–85, cognitively unimpaired, and had elevated levels of brain amyloid as measured by amyloid PET. The study was a 4.5‐year placebo‐controlled multisite trial of solanezumab with a 90‐day screening period (with a minimum of four visits) and monthly in‐clinic infusions for over 240 weeks.^14^ The A4 Study was conducted in accordance with International Council for Harmonisation Good Clinical Practice guidelines with approval from local institutional review boards at each participating site.

**RESEARCH IN CONTEXT**

1. **Systematic review**: The authors of this manuscript conducted a thorough review of existing literature using PubMed and other traditional sources on participant research satisfaction and participant retention in AD research. With the increasing number of preclinical AD trials, there is a corresponding need to provide evidence‐based data to guide trialists designing these trials. Relevant sources have been appropriately cited.
2. **Interpretation**: Overall, participants in the A4 Study were highly satisfied with their trial experience. Baseline differences in satisfaction were associated with study completion status, suggesting that the RSS may serve as a key tool to assess the risk of participant attrition. We identified trial aspects that participants strongly affirmed including aspects associated with altruistic, personal, and social motivations, while elucidating modifiable trial aspects that may warrant attention to enhance participant experience. Lastly, we also found that the number of trial aspects that participants identified as needing improvement may serve as an important indicator of participant retention.
3. **Future directions**: Further examination of participant research satisfaction and feedback is needed across different preclinical AD trials to determine the generalizability of our findings. Additionally, extending this research to MCI and later stage AD trials will be important to determine whether these findings remain consistent across disease stages.

### 2.2 RSS

The RSS (Supplemental Material 1) was administered at five time points (visit 6 [week 0/baseline/randomization visit], visit 18 [week 48], visit 33 [week 108], visit 48 [week 168], and visit 66 [week 240]) and the study discontinuation visit during the double‐blind phase of the trial. We focused on the first 7 items (Supplemental Material 1) in our analysis as the final question (Q8) was specific to the A4 trial (quality of the iPad testing) and not generalizable to other studies. We divided the RSS into the following three sections: (A) *Participant satisfaction with the trial* (Questions 1–4), where participants were asked to select one of four ordinal responses for each question; (B) *most favored and disfavored trial aspects* (Questions 5–6), where participants were asked to select one of seven pre‐specified trial aspects for each question; and (C) *trial aspects that the participants would like to change* (Question 7), where the available responses were binary (yes/no) for four pre‐specified trial aspects (study medication, test and/or procedures, visit schedule, and other).

### 2.3 Participant characteristics

Our analysis included data on participant demographics (age, sex, education, race, ethnicity, marital status), baseline clinical and cognitive assessment scores (Alzheimer's Disease Cooperative Study Preclinical Alzheimer's Cognitive Composite [PACC], Geriatric Depression Scale [GDS], State‐Trait Anxiety Inventory [STAI], Impact of Event Scale [IES], cognitive function index [CFI]), and other characteristics (study partner type, treatment group, family history of dementia, APOE e4 carrier status [not disclosed to participants]). We created a binary racial and ethnic underrepresented group (RE‐URG) variable due to the small sample of participants who self‐identified their race as not White or their ethnicity as Hispanic (< 10%). Individuals identifying as being of American Indian or Alaska Native race, Asian race, Black or African American race, more than one race, or of Hispanic ethnicity were considered as RE‐URG. Participants who reported as being of White race and not of Hispanic ethnicity were categorized as not a RE‐URG.

### 2.4 Statistical analysis

For this study, participants with an early termination visit were binned to the next scheduled visit. We outline the analytic approaches used for each section of the RSS.

#### 2.4.1 Sections (A) and (B)

For the purposes of our analysis, due to small sample sizes in a few response categories, we dichotomized the responses for Section (A) to two categories “satisfied” or “not satisfied” (details are described in Supplemental Material 2). For section (B), we derived mutually exclusive constructs from seven prespecified responses and the other responses. The most favored constructs included altruistic, personal, social, and mixed or other; the most disfavored constructs included design, site, individual expectations/fears, none, and mixed or other. Common themes were identified and reviewed before finalization by two investigators (M.R., R.R.). Detailed categorization criteria and description for the thematic constructs are outlined in Supplemental Material 3.

We evaluated the responses to each question at baseline and over time. For questions with sufficient variability in responses (defined as at least 5% of participants endorsing a non‐modal response), we descriptively examined participant characteristics associated with baseline RSS responses. Continuous variables were reported as means and standard deviations and categorical variables were reported as frequencies and percentages. Univariate generalized linear mixed models with site‐specific random effects were used to examine potential differences in baseline RSS responses between trial completers and non‐completers. Additionally, we conducted multivariable analyses adjusting the models for variables previously identified as pre‐randomization predictors of study discontinuation in the A4 study (age, STAI, family history of dementia, and apolipoprotein E [APOE] e4 status).^13^ Sensitivity analyses were conducted limiting the sample of non‐completers to those who voluntarily withdrew or were lost to follow up given that this reason of attrition may be more modifiable than discontinuation due to adverse events, medical conditions, or death.

#### 2.4.2 Section (C)

We evaluated the frequency and proportion of each response collected at the participants’ final or termination visit by study completion status. We also reported results limiting the sample to participants who voluntarily withdrew or were lost to follow up as sensitivity analyses. Lastly, we reported the frequency of distinct free‐text responses within the “other” category.

## 3 RESULTS

### 3.1 Section A: Participant satisfaction with the trial

Of the 1169 participants that were randomized in the A4 Study, 1161 participants completed at least one item on the RSS at baseline. Responses to RSS questions 1 to 4 prior to categorization are summarized in Supplemental Material 4. At this baseline visit, 1153 (99.4%) participants reported being “satisfied” with the quality of tests and attention received, 1136 (98.7%) with the extent to which the program met their expectations, 1147 (98.8%) with recommending the study to a friend, and 1070 (92.2%) with their decision to enroll again.

**FIGURE 1:**
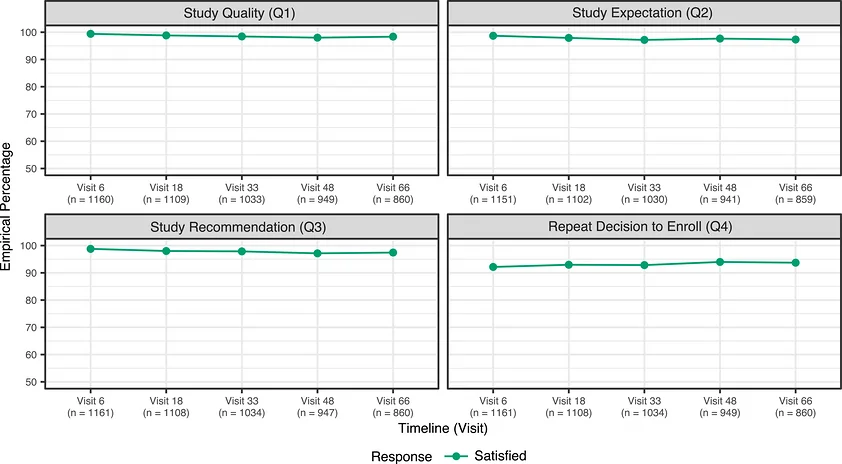
Proportion of participants who reported being “satisfied” (positive response) on overall research satisfaction (Q1–4 of RSS) at five time points throughout the A4 Study. A4 Study, Anti‐Amyloid Treatment for Asymptomatic AD Study; RSS, Research Satisfaction Survey; Q, question.

**FIGURE 2 alz71852-fig-0002:**
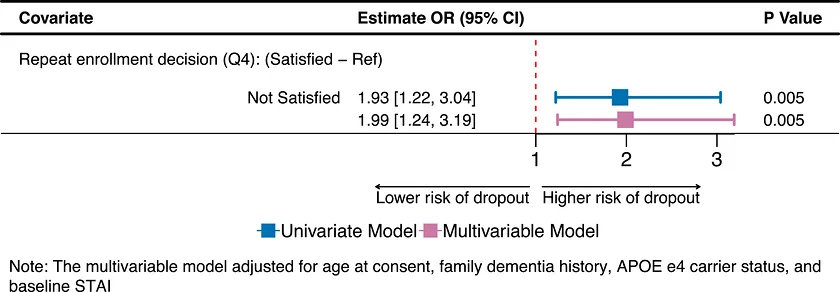
Trial completion status by baseline participant response to Q4 (repeating decision to enroll). Q, question.

Across each time point, over 90% of the participants reported being satisfied on all four research satisfaction questions (Figure 1). Subsequent analyses were not conducted for Q1–3 due to the small sample size of those who selected “not satisfied” (< 3% for each). For Q4 (whether the participant would repeat the decision to participate in the trial again), we examined the baseline responses by participant characteristics (Table 1) and by trial completion status (Figure 2). Participant demographic characteristics were similar across age, sex, education, RE‐URG status, and marital status. Overall, participants most frequently enrolled with spousal partners (64.3%) followed by other study partners (e.g., friends/companion, sibling, other relative) (23.2%) and adult children (12.5%). Among participants reporting satisfaction, a higher proportion enrolled with spousal partners compared to those who were not satisfied (65.1% vs., 54.9%), whereas a lower proportion enrolled with other partner types (22.9% vs 27.5%) or adult children (12.0% vs 17.6%). Baseline clinical and cognitive assessment scores (PACC, GDS, STAI, IES, CFI) were similar between those who were satisfied and those who were not. When examining potential differences in trial completion status by baseline Q4 response, we found that participants who would not repeat the decision to enroll had higher odds of early study discontinuation than those who would (multivariable odds ratio: 1.99; 95% confidence interval [CI]: 1.24, 3.19; *p* = 0.005). In a follow up analysis, we found that among those who discontinued early from the study 11.4% of participants reported that they would not repeat the decision to enroll again at baseline.

**TABLE 1:** Participant characteristics by baseline Q4 (repeat decision to enroll) response.

Characteristics	Satisfied (*N* = 1070)	Not satisfied (*N* = 91)	Total (*N* = 1161)
Female sex, *n* (%)	629 (58.8)	60 (65.9)	689 (59.3)
Age, mean (sd)	72.0 (4.8)	71.3 (5.0)	71.9 (4.8)
RE‐URG^*^, *n* (%)			
Not URG	989 (94.0)	84 (92.3)	1073 (93.9)
URG	63 (6.0)	7 (7.7)	70 (6.1)
Missing	18	0	18
Marital status, *n* (%)			
Married	774 (73.3)	57 (66.3)	831 (72.8)
Divorced	38 (3.6)	2 (2.3)	40 (3.5)
Never married	89 (8.4)	13 (15.1)	102 (8.9)
Widowed	155 (14.7)	14 (16.3)	169 (14.8)
Missing	14	5	19
Study partner type, *n* (%)			
Spouse	686 (65.1)	50 (54.9)	736 (64.3)
Adult child/child‐in‐law	127 (12.0)	16 (17.6)	143 (12.5)
Other	241 (22.9)	25 (27.5)	266 (23.2)
Missing	16	0	16
Family dementia history, *n* (%)	799 (74.7)	73 (80.2)	872 (75.1)
Education, *n* (%)			
≤ High school graduate	106 (9.9)	7 (7.7)	113 (9.7)
Some college degree	514 (48.0)	47 (51.6)	561 (48.3)
Professional degree	450 (42.1)	37 (40.7)	487 (41.9)
Treatment group, solanezumab, *n* (%)	532 (49.7)	39 (42.9)	571 (49.2)
APOE e4, carrier, n (%)	627 (58.6)	58 (63.7)	685 (59.0)
Baseline PACC^†^, mean (SD)	0.0 (2.7)	0.2 (2.7)	0.0 (2.7)
Baseline GDS^‡^, mean (SD)	1.1 (1.4)	1.3 (1.8)	1.1 (1.4)
Baseline STAI^§^, mean (SD)	9.8 (3.0)	10.9 (3.4)	9.9 (3.1)
Baseline IES^¶^, mean (SD)	10.0 (10.6)	13.1 (12.5)	10.2 (10.8)
Baseline CFI^#^, mean (SD)	2.4 (2.2)	2.3 (2.1)	2.4 (2.2)

Abbreviations: APOE, apolipoprotein E; Q, question; SD, standard deviation; URG, underrepresented group.

* RE‐URG: Racial and ethnic underrepresented group.

^†^ PACC: Alzheimer's Disease Cooperative Study Preclinical Alzheimer's Cognitive Composite.

^‡^ GDS: Geriatric Depression Scale.

^§^ STAI: State‐Trait Anxiety Inventory.

^¶^ IES: Impact of Event Scale.

^#^ CFI: Cognitive Function Index.

### 3.2 Section B: Most and least favored trial aspects

**FIGURE 3:**
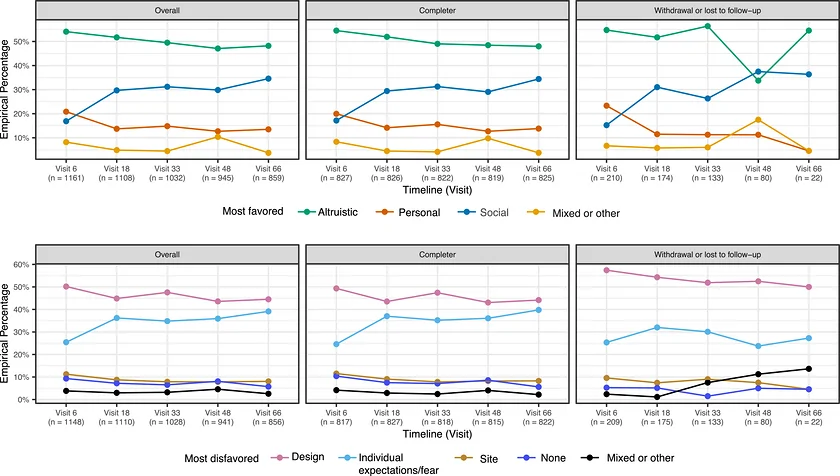
Trial constructs that participants most favored and disfavored across five time points by study completion status.

Baseline assessment of the most favorable aspects of trial participation indicated that the altruistic construct was most frequently endorsed (628 participants; 54.1%), followed by the personal construct (242; 20.8%), the social construct (196; 16.9%), and the mixed or other construct (95; 8.2%). Participants most frequently affirmed the altruistic construct at all time[Fig alz71852-fig-0002] points (Figure 3a). This construct included responses such as volunteering, raising awareness and contributing to science/a cure/helping others. Longitudinal trends over time showed that the proportion of participant endorsement of the social construct increased (bl: 196; 16.9%; visit 66: 297; 34.6%), while endorsement of the altruistic (bl: 628; 54.1%; visit 66: 414; 48.2%) and personal (bl: 242; 20.8%; visit 66: 116; 13.5%) constructs remained largely stable, with a slight decrease. When stratifying the results by trial completion status we observed similar patterns.

The most disfavored trial aspect at baseline was the design construct (576 participants; 50.2%) followed by the individual expectations/fears construct (292; 25.4%), the site construct (129; 11.2%), the none construct (107; 9.3%), and the mixed or other construct (44; 3.8%). The design construct was the most disfavored trial aspect at all time points, though we observed a downward trend in the proportion of participants selecting this construct over time (bl: 576; 50.2%; visit 66: 381; 44.5%). In contrast, the proportion of responses for the individual expectations/fear construct as disfavored was higher at visit 66 (335; 39.1%) compared to baseline (292; 25.4%) (Figure 3b). When stratified by completion status, we observed that completers more often reported no dislikes (83; 10.3%) at baseline, compared to those who withdrew or were lost to follow‐up (9; 5.3%). Among participants who withdrew or were lost to follow‑up, 22 had a completed RSS response at visit 66. Within this group, the proportion of participants selecting the mixed or other construct was higher at visit 66 (13.6% of 22 participants), than at baseline (2.4% of 209 participants), while the proportions remained relatively consistent among completers (bl: 4.2% of 817 participants; visit 66: 2.6% of 822 participants) (Figure 3b). Given the small number of participants remaining at visit 66, these descriptive differences should be interpreted with caution.

Baseline Q5 (most strongly affirmed trial aspect) responses varied by participant characteristics (Table 2). A higher proportion of female participants (62.3%) selected the altruistic construct compared to the personal (55.0%), social (58.7%), and mixed or other (52.6%) constructs. Participants belonging to a RE‐URG made up 14% of responses associated with the personal construct compared to 6.2%, 7.3%, and 7.3% of responses associated with the altruistic, social, and mixed or other constructs, respectively. For the most disfavored trial aspects (Q6, Table 3), female participants accounted for 69.0% of responses associated with the site construct compared to 59.7%, 59.2%, 56.8%, and 50.5% of responses associated with the design, individual expectations/fears, mixed or other, and none constructs, respectively. Participants belonging to a RE‐URG accounted for 9.3% of the mixed or other construct and 8.4% of the none construct compared to 6.3% of the design, 4.9% of the individual expectations/fears, and 3.1% of the site constructs. Participants with a family history of dementia made up 86.4% of the mixed or other, 81.4% of the site, 75.7% of the design, 71.0% of the none, and 70.5% of the individual expectations/fears constructs. We also observed variation by education where 11.6% of individuals with less than or equal to high school education reported having “no dislikes” compared to 10.4% among those with some college degree and 7.5% among those with professional degrees.

**TABLE 2:** Participant characteristics by baseline Q5 (most favored trial aspect) response

Characteristics	Altruistic (*N* = 628)	Personal (*N* = 242)	Social (*N* = 196)	Mixed or other (*N* = 95)	Total (*N* = 1161)
Female sex, *n* (%)	391 (62.3)	133 (55.0)	115 (58.7)	50 (52.6)	689 (59.3)
Age, mean (sd)	71.5 (4.8)	72.3 (4.9)	72.7 (4.7)	72.3 (5.1)	71.9 (4.8)
RE‐URG^*^, *n* (%)					
Not URG	584 (94.3)	220 (91.7)	183 (94.8)	86 (94.5)	1073 (93.9)
URG	35 (5.7)	20 (8.3)	10 (5.2)	5 (5.5)	70 (6.1)
Missing	9	2	3	4	18
Marital status, *n* (%)					
Married	453 (73.5)	167 (69.9)	146 (74.9)	65 (70.7)	831 (72.8)
Divorced	20 (3.2)	7 (2.9)	9 (4.6)	4 (4.3)	40 (3.5)
Never married	50 (8.1)	23 (9.6)	18 (9.2)	11 (12.0)	102 (8.9)
Widowed	93 (15.1)	42 (17.6)	22 (11.3)	12 (13.0)	169 (14.8)
Missing	12	3	1	3	19
Study partner type, *n* (%)					
Spouse	396 (64.2)	148 (62.2)	132 (67.3)	60 (63.8)	736 (64.3)
Adult child/child‐in‐law	80 (13.0)	33 (13.9)	15 (7.7)	15 (16.0)	143 (12.5)
Other	141 (22.9)	57 (23.9)	49 (25.0)	19 (20.2)	266 (23.2)
Missing	11	4	0	1	16
Family dementia history, *n* (%)	482 (76.8)	181 (74.8)	142 (72.4)	67 (70.5)	872 (75.1)
Education, *n* (%)					
≤ High school graduate	69 (11.0)	23 (9.5)	12 (6.1)	9 (9.5)	113 (9.7)
Some college degree	306 (48.7)	115 (47.5)	100 (51.0)	40 (42.1)	561 (48.3)
Professional degree	253 (40.3)	104 (43.0)	84 (42.9)	46 (48.4)	487 (41.9)
Treatment group, solanezumab, *n* (%)	295 (47.0)	127 (52.5)	106 (54.1)	43 (45.3)	571 (49.2)
APOE e4, carrier, *n* (%)	382 (60.8)	135 (55.8)	111 (56.6)	57 (60.0)	685 (59.0)
Baseline PACC^†^, mean (SD)	0.2 (2.6)	−0.2 (2.8)	−0.4 (2.7)	−0.2 (2.6)	0.0 (2.7)
Baseline GDS^‡^, mean (SD)	1.0 (1.3)	1.1 (1.6)	1.2 (1.7)	1.1 (1.2)	1.1 (1.4)
Baseline STAI^§^, mean (SD)	9.8 (2.9)	10.1 (3.2)	9.8 (3.3)	10.4 (3.2)	9.9 (3.1)
Baseline IES^¶^, mean (SD)	9.4 (10.0)	11.5 (10.6)	11.2 (12.3)	10.4 (12.1)	10.2 (10.8)
Baseline CFI^#^, mean (SD)	2.2 (2.0)	2.7 (2.2)	2.4 (2.2)	2.7 (2.6)	2.4 (2.2)

Abbreviations: APOE, apolipoprotein E; Q, question; SD, standard deviation; URG, underrepresented group.

* RE‐URG: Racial and ethnic underrepresented group.

^†^ PACC: Alzheimer's Disease Cooperative Study Preclinical Alzheimer's Cognitive Composite.

^‡^ GDS: Geriatric Depression Scale.

^§^ STAI: State‐Trait Anxiety Inventory.

^¶^ IES: Impact of Event Scale.

^#^ CFI: Cognitive Function Index.

**TABLE 3:** Participant characteristics by baseline Q6 (most disfavored trial aspect) response.

Characteristics	Design (*N* = 576)	Individual expectations/fears (*N* = 292)	Site (*N* = 129)	None (*N* = 107)	Mixed or other (*N* = 44)	Total (*N* = 1148)
Female sex, *n* (%)	344 (59.7)	173 (59.2)	89 (69.0)	54 (50.5)	25 (56.8)	685 (59.7)
Age, mean (SD)	72.0 (4.9)	72.1 (4.7)	71.6 (4.7)	71.9 (4.9)	71.5 (4.5)	71.9 (4.8)
RE‐URG^*^, *n* (%)						
Not URG	532 (93.7)	270 (95.1)	124 (96.9)	98 (91.6)	39 (90.7)	1063 (94.1)
URG	36 (6.3)	14 (4.9)	4 (3.1)	9 (8.4)	4 (9.3)	67 (5.9)
Missing	8	8	1	0	1	18
Marital status, *n* (%)						
Married	420 (74.1)	205 (71.9)	88 (68.2)	79 (74.5)	30 (69.8)	822 (72.7)
Divorced	18 (3.2)	10 (3.5)	9 (7.0)	3 (2.8)	0 (0.0)	40 (3.5)
Never married	46 (8.1)	27 (9.5)	10 (7.8)	12 (11.3)	5 (11.6)	100 (8.8)
Widowed	83 (14.6)	43 (15.1)	22 (17.1)	12 (11.3)	8 (18.6)	168 (14.9)
Missing	9	7	0	1	1	18
Study partner type, *n* (%)						
Spouse	372 (65.3)	180 (62.7)	84 (65.1)	65 (62.5)	26 (60.5)	727 (64.2)
Adult child/child‐in‐law	64 (11.2)	45 (15.7)	15 (11.6)	11 (10.6)	8 (18.6)	143 (12.6)
Other	134 (23.5)	62 (21.6)	30 (23.3)	28 (26.9)	9 (20.9)	263 (23.2)
Missing	6	5	0	3	1	15
Family dementia history, *n* (%)	436 (75.7)	206 (70.5)	105 (81.4)	76 (71.0)	38 (86.4)	861 (75.0)
Education, *n* (%)						
≤ High school graduate	65 (11.3)	22 (7.5)	8 (6.2)	13 (12.1)	4 (9.1)	112 (9.8)
Some college degree	275 (47.7)	148 (50.7)	55 (42.6)	58 (54.2)	17 (38.6)	553 (48.2)
Professional degree	236 (41.0)	122 (41.8)	66 (51.2)	36 (33.6)	23 (52.3)	483 (42.1)
Treatment group, solanezumab, *n* (%)	274 (47.6)	148 (50.7)	63 (48.8)	55 (51.4)	24 (54.5)	564 (49.1)
APOE e4, carrier, *n* (%)	339 (58.9)	173 (59.2)	82 (63.6)	62 (57.9)	23 (52.3)	679 (59.1)
Baseline PACC^†^, mean (SD)	−0.0 (2.8)	0.0 (2.6)	0.2 (2.7)	−0.3 (2.5)	0.7 (2.4)	0.0 (2.7)
Baseline GDS^‡^, mean (SD)	1.0 (1.4)	1.3 (1.6)	1.1 (1.5)	0.8 (0.9)	1.1 (1.4)	1.1 (1.4)
Baseline STAI^§^, mean (SD)	9.9 (3.0)	10.1 (3.1)	9.9 (3.1)	9.0 (2.6)	10.7 (3.4)	9.9 (3.1)
Baseline IES^¶^, mean (SD)	10.0 (10.2)	12.0 (12.0)	8.4 (9.4)	8.3 (10.0)	13.3 (12.7)	10.3 (10.8)
Baseline CFI^#^, mean (SD)	2.3 (2.2)	2.4 (2.0)	2.4 (2.3)	2.3 (2.3)	2.5 (2.5)	2.4 (2.2)

Abbreviations: APOE, apolipoprotein E; Q, question; SD, standard deviation; URG, underrepresented group.

* RE‐URG: Racial and ethnic underrepresented group.

^†^ PACC: Alzheimer's Disease Cooperative Study Preclinical Alzheimer's Cognitive Composite.

^‡^ GDS: Geriatric Depression Scale.

^§^ STAI: State‐Trait Anxiety Inventory.

^¶^ IES: Impact of Event Scale.

^#^ CFI: Cognitive Function Index.

**FIGURE 4:**
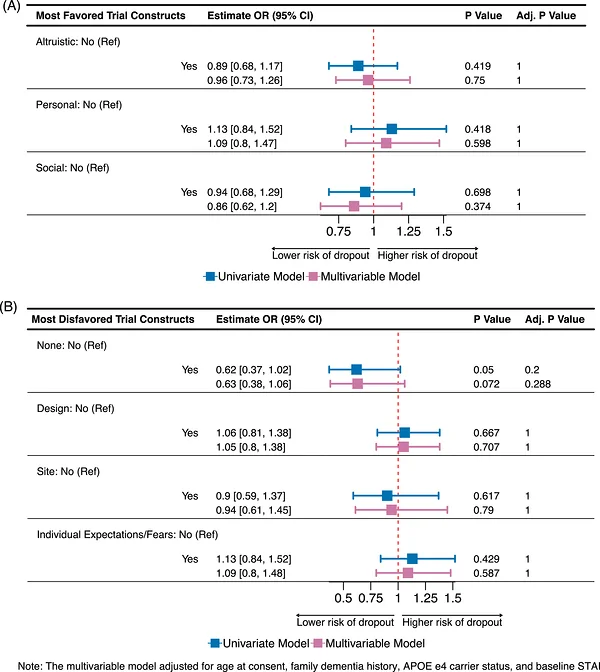
Trial completion status by (A) Q5 (most favored construct) and (B) Q6 (most disfavored construct) responses at baseline. Q, question.

In our analysis of study discontinuation risk, we found no significant differences by baseline responses to the most favored (Q5) and disfavored (Q6) trial constructs (Figure 4).

### 3.3 Section C: Trial aspects that the participants would like to change

At the participants’ final visit (end of study or early termination), most participants indicated no desire to change the study medication (1004 participants; 92.5%), test and/or procedures (954; 87.8%), visit schedule (1009; 92.8%), and other trial aspects (822; 83.5%) (Table 4). A higher proportion of participants who discontinued the trial early reported “yes” to changing all 4 trial aspects compared to study completers. The results were consistent when limiting the sample to participants who voluntarily withdrew or were lost to follow up among the non‐completers. In our sensitivity analysis, we found that the most reported response under the “other” category was “lack of feedback”, reported by both completers (50.4%; *n* = 58) and those who withdrew or were lost to follow up (45.9%; *n* = 17). The second most frequent response was “travel to site/parking” reported by 13.0% (*n* = 15) of completers and 18.9% (*n* = 7) of participants who withdrew or were lost to follow‐up. All other categorized free text responses were selected by less than 10 participants.

**TABLE 4:** Trial aspects that participants would like to change (Q7) reported at final visit

Parameter	Non‐completers (*N* = 339)	Completers (*N* = 830)	Total (*N* = 1169)
Q7a. Study medication			
No	230 (84.6%)	774 (95.2%)	1004 (92.5%)
Yes	42 (15.4%)	39 (4.8%)	81 (7.5%)
Missing	67	17	84
Q7b. Test and/or procedures			
No	236 (87.1%)	718 (88.0%)	954 (87.8%)
Yes	35 (12.9%	98 (12.0%)	133 (12.2%)
Missing	68	14	82
Q7c. Number of visits			
No	239 (87.9%)	770 (94.5%)	1009 (92.8%)
Yes	33 (12.1%)	45 (5.5%)	78 (7.2%)
Missing	67	15	82
Q7d. Other			
No	184 (78.0%)	638 (85.3%)	822 (83.5%)
Yes	52 (22.0%)	110 (14.7%)	162 (16.5%)
Missing	103	82	185

*Note*: Responses collected at visit 66 were used for participants who completed the blinded phase of the study. Responses collected at either the early discontinuation visit or any last known visit (other than baseline visit [visit 6]) were used for participants who discontinued during the blinded phase of the study.

Abbreviation: Q, question.

## 4 DISCUSSION

The current study is among the first to systematically examine participant research satisfaction among participants enrolled in a large multi‐site preclinical AD trial. Overall, satisfaction with the 4.5‐year A4 Study was uniformly high and consistent across time. Differences in satisfaction were observed among those who completed the trial, compared to those who withdrew participation prior to completion.

The baseline RSS data collected at the first post‐screening visit revealed that 92% of participants would again choose to participate in the trial if given the chance to re‐evaluate their decision. More than 98% of participants reported being satisfied with the quality of the tests and attention they received, felt the research program met their expectations, and were likely to recommend the study to a friend at baseline. While it is possible that participant enthusiasm may have been partly enhanced by the novelty of the A4 Study (as the first multisite trial intervening in the preclinical stage of AD), these results offer encouraging evidence for the acceptability of the protocol and the level of commitment required from participants in preclinical AD trials. We observed some variability in baseline responses to Q4 (willingness to participate in the study again), which were based solely on participants’ impressions from the 90‐day, 4‐visit screening process. Although the sample of unsatisfied responses was small (8%), the screening experience alone, without exposure to other trial components such as regular infusions, reduced some individuals’ willingness to repeat their decision to enroll.

Participant responses to Q4 suggested a higher risk of attrition among individuals who reported lower satisfaction at baseline compared with those who expressed higher satisfaction. This finding should be interpreted with caution as the precision of the estimate was limited, as reflected by the wide confidence interval around the odds ratio. Nonetheless, responses to the question regarding willingness to enroll again may serve as an informative indicator of subsequent retention. Additional data collected from future preclinical AD trials will help determine the robustness of this relationship and its potential utility for informing retention strategies. While we did not find differences in Q4 responses by demographic and clinical characteristics, we observed that among those who reported being satisfied, a higher proportion enrolled with spousal study partners and a lower proportion enrolled with adult children or other study partners compared to those who were not satisfied. This may suggest that the current study designs are better aligned with the needs of spousal dyads, underscoring the need to reduce barriers faced by participants who enroll with non‐spousal study partners (e.g. offering transportation resources, flexible scheduling times, increasing compensation etc.).^15^

Prior survey research among older adults enrolled in longitudinal studies of aging (without information on clinical diagnoses), has shown that decisions about continued participation are shaped by multiple factors, most notably altruistic motivations, concerns about health, and the value participants’ place on their relationship with experts.^16^ Similarly, a recently completed nationally representative survey of older adults in the United States demonstrated that messages around participation benefits and altruistic motivations effectively increased enrollment intention in AD registries.^17^ We extended these findings to a clinical trial context among individuals with preclinical AD. In this setting, we found that the most frequently affirmed trial aspects overall were associated with altruistic motivations, consistent with existing literature.^18^, ^19^, ^20^, ^21^ The personal construct represented the second most frequently affirmed trial aspect at baseline and largely included concerns about personal health. While altruistic and personal constructs remained highly cited throughout the trial, we observed a marked increase in responses associated with social motivations which displaced the personal construct as the second most affirmed construct at visit 18 (second time point) and onward. This temporal change aligns with, and builds upon, prior research highlighting the importance of participant‐staff interactions in shaping participant trial experience.^12^, ^18^ Interpretation of these findings should also consider the overlap of the study period with the coronavirus disease 2019 (COVID‐19) pandemic, although the extent to which pandemic‐related factors influenced social motivations remain unclear. When stratifying the outcomes by trial completion status, the changes over time in the social construct were even more pronounced among those who withdrew or were lost to follow‐up. While social motivations may not fully offset the barriers to continued participation, they represent important motivators that potentially compensate for the declining satisfaction with other trial aspects. Strengthening support for site staff, participants, and study partners through the implementation of engagement efforts may therefore serve as an effective retention strategy.

When examining trial elements that participants least favored, several key patterns emerged. Firstly, we found that responses associated with study design were most frequently indicated as disfavored throughout the trial. Decisions around study design, however, are complex.^22^, ^23^ Our previous work demonstrated that trial duration impacts the odds of study completion^24^, yet preclinical AD trials typically require longer duration compared to trials of later AD stages due to the slower changes in measurable outcomes.^25^ While similar to the constraints of trial duration, the design construct included several trial aspects that were essential for the scientific validity of the A4 Study (e.g., blinding of randomization, the study medication, mode of administration), future trials may have opportunities to introduce flexibility in areas that were not modifiable in the present study. For example, investigators may consider options such as remote or alternative testing/procedures, reduced in‐clinic time, or decreased repetitiveness of assessments to help minimize participant burden. Furthermore, compared to trial completers, the proportion of participants who selected the design construct was higher among those who withdrew or were lost to follow up, indicating that study design features not only affect participant satisfaction but may also influence decisions around continued participation. The second most frequently cited response throughout the trial was associated with the individual expectations/fear construct, which included responses such as “lack of feedback”, “feeling inadequate”, and “reminder/fear of AD risk”. The proportion of participants selecting this construct also increased throughout the study, suggesting a potential discrepancy between initial expectations at enrollment and actual study experience. These findings are in line with a previous survey study among AD research center enrollees that found that the most common suggestion to improve study experience was to provide more feedback and opportunities for learning about AD.^26^ While investigators are generally unable to provide trial related results to participants until study completion, offering reassuring messages of support, feedback on trial progress, recent updates in AD research, and individual participant milestones, may enhance participant engagement and satisfaction, thereby increasing retention. These findings have informed the development of novel retention strategies such as non‐unblinding participant webinar series and more enhanced participant communication implemented in the AHEAD 3–45 Study^2^, an ongoing multi‐site preclinical AD trial. Lastly, we found that the proportion of participants who selected mixed or other responses increased over time among those who withdrew or were lost to follow up (Figure 3b), suggesting that those who discontinued at later timepoints reported a broader set of concerns than those who withdrew early. Among study completers, the selection of this construct remained consistently low. The number of trial aspects that participants identify as needing improvement therefore may serve as an important indicator of participant retention that should be periodically assessed.

Consistent with responses associated with research satisfaction (Section A), our analysis of trial aspects that participants would like to change (Section C) yielded largely positive responses, with over 80% indicating no desire to change the study medication, test and/or procedures, number of visits, and other trial aspects. The “other” category had the highest proportion of “yes” responses (16.5%), approximately half of which were free‐text entries identifying “lack of feedback” as the trial aspect they would like to change. For all predefined trial aspects, the proportion of participants who desired change was higher among those who discontinued the trial prematurely, reinforcing the potential utility of the RSS to inform future retention strategies.

This study was not without limitations. The A4 study encountered disruptions to the planned testing and dose administration schedules due to the COVID‐19 pandemic. Nevertheless, participant satisfaction remained high, with only 10 participants citing the pandemic as the primary reason for study withdrawal.^27^ While the RSS items were designed with forced‐choice response options, a high proportion of participants selected multiple responses thereby limiting our ability to identify a single reason. The study findings may have limited generalizability given the small sample size of participants from underrepresented demographic groups (e.g., underrepresented racial and ethnic groups, rural residents, those of lower socioeconomic status). Future analyses of participant feedback will be important in other phase III preclinical AD trials.^2^, ^3^ Lastly, we created larger constructs for the purposes of our analysis, but individual free‐text responses may need to be examined to identify nuanced retention strategies for future preclinical AD trials.

## 5 CONCLUSION

By using the RSS to capture participant feedback throughout the trial, this study sheds new light on participant experiences in a preclinical AD trial and highlights the value of such feedback in guiding future trial conduct and study design choices. The overwhelming majority of participants reported having a positive experience in this long duration trial with extensive testing procedures and frequent infusions conducted during the global COVID‐19 pandemic. Differences in RSS responses were observed between trial completers and non‐completers at baseline and may serve as an important tool to identify those at risk of premature study discontinuation. Retention strategies that respond to concerns about design burden and lack of feedback as well as those that increase social engagement will be important for investigators to prioritize. These insights can guide and instruct modifiable study design elements, strengthen retention strategies, and improve trial processes to better align with participant needs and value.

## HUMAN STUDIES

Each A4 Study site obtained approval from their local institutional review board, and the study was performed in accordance with the ethical standards as laid down in the 1964 Declaration of Helsinki and its later amendments or comparable ethical standards.

## CONFLICT OF INTEREST STATEMENT

M.R. has received funding from the Alzheimer's Clinical Trial Consortium and the American Heart Association. O.L. has received grant funding to the Institution from Eisai, and the National Institutes of Health. MCD's spouse is a fulltime employee of Johnson & Johnson. J.D.G. has received funding from NIA, Alzheimer's Association, BrightFocus Foundation, Eli Lilly, Genentech, Biogen, and Eisai. He reports consulting for Valuate Health, travel provision from the Alzheimer's Association, and payment for editorial service to Alzheimer's & Dementia. R.A.S. has served as a consultant for AbbVie, AC Immune, Acumen, Alector, Apellis, Biohaven, Bristol Myers Squibb, Genentech, ImmunoBrain, Ionis, Janssen, Oligomerix, Prothena, Roche, and Vaxxinity over the past 3 years. She has received research funding from Eisai and Eli Lilly for public‐private partnership clinical trials and receives research grant funding from the National Institute on Aging/National Institutes of Health, GHR Foundation, and the Alzheimer's Association. Her spouse, K. Johnson, reports consulting fees from Novartis, Merck, and Janssen. R.R. has received funding from NIA, Alzheimer's Association, Alzheimer's Association, American Heart Association and Eisai. Other authors declare that they have no competing interests. Author disclosures are available in the Supporting Information.

## CONSENT

The participants in the A4 Study consented to share their de‐identified data for secondary analyses such as those reported in this study.

## Supporting information




Supporting Information

Supporting Information

Supporting Information

Supporting Information

Supporting Information
