# Supplementary material for: Research satisfaction among participants in a preclinical Alzheimer's disease trial
Source: Alzheimers Dement. 2026 Sep 22;22(9):e71852. doi: 10.1002/alz.71852 (PMC13597467; doi:10.1002/alz.71852)
Supplement: Supplementary file 2 [file ALZ-22-e71852-s002.pdf]

Supplemental Material 2: Response categorization for research satisfaction items (Q1-4) on the RSS

Question	Derived categorization	Available response
1. How would you rate the quality of the tests and attention you have received during your participation in the study?	Satisfied	• Excellent<br>• Good
	Not satisfied	• Fair<br>• Poor
2. To what extent has our research program met your expectations?	Satisfied	• Almost all of my expectations have been met<br>• Most of my expectations have been met
	Not satisfied	• Only a few of my expectations have been met<br>• None of my expectations have been met
3. If a friend were to inquire about this research program, would you recommend it to him or her?	Satisfied	• Yes, generally<br>• Yes, definitely
	Not satisfied	• No, definitely not<br>• No, not really
4. If you had a chance to redo your decision to participate in this research program, do you think you would choose to participate?	Satisfied	• Yes, definitely<br>• Yes, I think so
	Not satisfied	• No, definitely not<br>• No, I don't think so
