# Supplementary material for: Research satisfaction among participants in a preclinical Alzheimer's disease trial
Source: Alzheimers Dement. 2026 Sep 22;22(9):e71852. doi: 10.1002/alz.71852 (PMC13597467; doi:10.1002/alz.71852)
Supplement: Supplementary file 3 [file ALZ-22-e71852-s003.pdf]

Supplemental Material 3: Construct categorization for items Q5 (most favored trial aspect) and Q6 (most disfavored trial aspect) of the RSS. **Blue** indicates the pre-specified responses on the RSS and **green** indicates derived subcategories from the free-text 'Other' responses.

**Most favored trial aspect (Q5)**		
Constructs	Description	Responses on the RSS
Altruistic	References expressing a desire to help others, advance science, contribute to public health, or benefit future generations	• Volunteering<br>• Raising awareness<br>• Contributing to science/a cure/helping others
Personal	References emphasizing personal health benefits, including health monitoring, information seeking, access to resources, and overall health improvement.	• Feedback on how I am doing<br>• Improves my mental function<br>• Possible benefit of treatment<br>• Education/learning<br>• Professional attention (e.g., monitoring, evaluation)
Social	References highlighting personal and social connection, community belonging, and enjoyment from engagement.	• Interacting with staff<br>• It was fun, engaging
Mixed or other	References including two or more motivational themes and those that did not align with a predefined construct	• More than one response<br>• Other
**Most disfavored trial aspect (Q6)**		
Constructs	Description	Responses on the RSS
Design	References to study design related considerations such as procedural or operational factors	• Time commitment<br>• Testing and/or procedures<br>• Repetitiveness<br>• The study medication<br>• Mode of administration/infusion problems<br>• Blinding of randomization (uncertainty of treatment group)
Site	References to study site specific logistical aspects of participation, including visit coordination, scheduling, delays and waiting time	• Travel to sites/parking<br>• Appointment scheduling<br>• Delays/waiting time
Individual expectations/fear	References to individual-level expectations or internal perceptions related to study participation, including trial expectations, self-assessment, and concerns related to AD risk	• Lack of feedback<br>• Feeling inadequate<br>• Reminder/fear of AD risk
None	No identification of most disfavored trial aspect, or explicit report of no dislikes.	• No dislikes
Mixed or other	References including two or more thematic areas or those that did not align with a predefined construct	• More than one response<br>• Other
