# Supplementary material for: Research satisfaction among participants in a preclinical Alzheimer's disease trial
Source: Alzheimers Dement. 2026 Sep 22;22(9):e71852. doi: 10.1002/alz.71852 (PMC13597467; doi:10.1002/alz.71852)
Supplement: Supplementary file 4 [file ALZ-22-e71852-s004.pdf]

Supplemental Material 1: Research Satisfaction Survey

This questionnaire is designed to evaluate your satisfaction with participation in the study. Only **one** answer per question should be marked.

1. **How would you rate the quality of the tests and attention you have received during your participation in the study?**

Excellent	Good	Fair	Poor

2. **To what extent has our research program met your expectations?**

Almost all of my expectations have been met	Most of my expectations have been met	Only a few of my expectations have been met	None of my expectations have been met

3. **If a friend were to inquire about this research program, would you recommend it to him or her?**

No, definitely not	No, not really	Yes, generally	Yes, definitely

4. **If you had a chance to redo your decision to participate in this research program, do you think you would choose to participate?**

No, definitely not	No, I don't think so	Yes, I think so	Yes, definitely

5. **What do you like best about the study?**
NOTE: Please choose only one response.

Volunteering	Interacting with staff	Feedback on how I am doing
It was fun, engaging	Improves my mental function	Raising awareness
Other, Specify:		

6. **What do you like least?**
NOTE: Please choose only one response.

Time commitment	Testing and/or procedures	Repetitiveness
Feeling inadequate	Lack of feedback	The study medication
Other, Specify:		

7. What would you change?

Study Medication	YES<br>NO	If YES, explain:
Testing/Procedures	YES<br>NO	If YES explain
Number of visits	YES<br>NO	If YES explain
Other	YES<br>NO	If YES explain

8. How would you rate the quality of the iPad Testing?

Excellent	Good	Fair	Poor
