# Supplementary material for: Research satisfaction among participants in a preclinical Alzheimer's disease trial
Source: Alzheimers Dement. 2026 Sep 22;22(9):e71852. doi: 10.1002/alz.71852 (PMC13597467; doi:10.1002/alz.71852)
Supplement: Supplementary file 5 [file ALZ-22-e71852-s005.pdf]

Supplemental Material 4: Responses to research satisfaction items (Q1-4) of the RSS prior to categorization across five time points.

	Visit-6 (N=1165)	Visit-18 (N=1074)	Visit-33 (N=982)	Visit-48 (N=904)	Visit-66 (N=830)	Visit-999 (N=191)
**1. How would you rate the quality of the tests and attention you have received during your participation in the study?**						
Missing	5	3	5	6	4	12
Excellent	961 (82.8%)	887 (82.8%)	786 (80.5%)	724 (80.6%)	648 (78.5%)	134 (74.9%)
Good	192 (16.6%)	172 (16.1%)	176 (18.0%)	156 (17.4%)	164 (19.9%)	42 (23.5%)
Fair	7 (0.6%)	12 (1.1%)	15 (1.5%)	17 (1.9%)	14 (1.7%)	2 (1.1%)
Poor	0 (0.0%)	0 (0.0%)	0 (0.0%)	1 (0.1%)	0 (0.0%)	1 (0.6%)
**2. To what extent has our research program met your expectations?**						
Missing	14	10	8	11	5	15
Almost all of my expectations have been met	840 (73.0%)	792 (74.4%)	691 (70.9%)	596 (66.7%)	558 (67.6%)	86 (48.9%)
Most of my expectations have been met	296 (25.7%)	254 (23.9%)	260 (26.7%)	279 (31.2%)	245 (29.7%)	74 (42.0%)
Only a few of my expectations have been met	12 (1.0%)	16 (1.5%)	18 (1.8%)	17 (1.9%)	21 (2.5%)	12 (6.8%)
None of my expectations have been met	3 (0.3%)	2 (0.2%)	5 (0.5%)	1 (0.1%)	1 (0.1%)	4 (2.3%)
**3. If a friend were to inquire about this research program, would you recommend it to him or her?**						
Missing	4	4	4	8	4	12
No, definitely not	4 (0.3%)	2 (0.2%)	2 (0.2%)	2 (0.2%)	4 (0.5%)	5 (2.8%)
No, not really	10 (0.9%)	16 (1.5%)	16 (1.6%)	20 (2.2%)	16 (1.9%)	10 (5.6%)
Yes, generally	296 (25.5%)	213 (19.9%)	193 (19.7%)	205 (22.9%)	169 (20.5%)	65 (36.3%)
Yes, definitely	851 (73.3%)	839 (78.4%)	767 (78.4%)	669 (74.7%)	637 (77.1%)	99 55.3%)
**4. If you had a chance to redo your decision to participate in this research program, do you think you would choose to participate?**						
Missing	4	3	4	6	4	13
No, definitely not	58 (5.0%)	39 (3.6%)	28 (2.9%)	28 (3.1%)	28 (3.4%)	9 (5.1%)
No, I don't think so	33 (2.8%)	27 (2.5%)	31 (3.2%)	19 (2.1%)	22 (2.7%)	32 (18.0%)
Yes, I think so	192 (16.5%)	174 (16.2%)	147 (15.0%)	153 (17.0%)	107 (13.0%)	48 (27.0%)
Yes, definitely	878 (75.6%)	831 (77.6%)	772 (78.9%)	698 (77.7%)	669 (81.0%)	89 (50.0%)
